# Point accuracy and reliability of an interstitial continuous glucose monitoring device in critically ill patients

**DOI:** 10.1186/cc14450

**Published:** 2015-03-16

**Authors:** R Van Hooijdonk, JH Leopold, T Winters, JM Binnekade, NP Juffermans, J Horn, JC Fischer, EC Van Dongen-Lases, MJ Schultz

**Affiliations:** 1 Academic Medical Center, Amsterdam, the Netherlands

## Introduction

There is a need for continuous glucose monitoring in critically ill patients. The objective of this trial was to determine the point accuracy and reliability of a device designed for continuous monitoring of interstitial glucose levels in ICU patients (Sentrino; Medtronic MiniMed, Northridge, CA, USA).

## Methods

Critically ill patients with an anticipated life expectancy >96 hours were eligible for participation, if the platelet count was >30 × 10^12 ^ml. Device readings were compared with glucose measurements in arterial blood using blood gas analyzers (RapidLab Siemens Healthcare Diagnostics, The Hague, the Netherlands). We used a linear mixed model to determine which factors affect point accuracy. In addition, we determined the reliability, including duration of device start-up and calibration, skips in data acquisition, and premature disconnections of sensors.

## Results

We included 50 patients, aged 65 (56 to 72) years with an APACHE II score of 23 (17 to 26). Admission types were medical (62%), elective surgery (22%) and emergency surgery (16%), and 22% had diabetes. For the accuracy analyses we had 929 comparative samples from 100 sensors in 45 patients (11 (7 to 28) samples per patient) during 4,639 hours (46 (27 to 134) hours per patient and 46 (21 to 69) hours per sensor). The Bland-Altman plot showed a bias of -0.6 mg/dl with limit of agreement between -57.2 and 56 mg/dl. Glucose prediction error analysis showed 60% of the glucose values <75 mg/dl within ±15 mg/dl and 75.8% of the glucose values ≥75 mg/dl within 20% of the comparative RapidLab results. Clarke error grid analysis showed 75.3% in zones A and 23.5% of the paired measurements in zones B, 0.3% of the paired measurements in zones C and 0.9% of the paired measurements in zones D. Point accuracy did not meet the ISO14971 standard for dosing accuracy, but improved with increasing numbers of calibrations, and was better in patients who did not have diabetes mellitus. Sixty out of 105 sensors were removed prematurely for a variety of reasons. The device start-up time was 49 (43 to 58) minutes. The number of skips in data acquisition was low, resulting in availability of real-time data during 95 (89 to 98)% of the connection time per sensor.

## Conclusion

The point accuracy of the device was relatively low in critically ill patients. The device reliability was relatively good, although sensors were removed prematurely for a variety of reasons.

